# Meta-analysis: long/short-term efficacy of anti-VEGF vs. panretinal photocoagulation in preventing severe complications in proliferative diabetic retinopathy

**DOI:** 10.1186/s40942-025-00687-0

**Published:** 2025-07-09

**Authors:** Tiago N. O. Rassi, Lucas M. Barbosa, Dillan Cunha Amaral, Ricardo N. Louzada, Helvécio N. F. Filho, Guilherme N. Marques, Breno C. Vieira, Sobha Sivaprasad, Mauricio Maia

**Affiliations:** 1 Hospital Fundação Banco de Olhos de Goiás, Setor de Retina e Vítreo, Goiânia, GO Brazil; 2 Departamento de Oftalmologia, Hospital VER Excelência em Oftalmologia, Universidade Federal de São Paulo, 260 Americano do Brasil Avenue, São Paulo, SP Brazil; 3 Faculdade de Medicina, Universidade Federal de Minas Gerais, Belo Horizonte, MG Brazil; 4 Faculdade de Medicina, Universidade Federal do Rio de Janeiro, Rio de Janeiro, RJ Brazil; 5 Faculdade de Medicina, Universidade de Fortaleza, Fortaleza, Ceará Brazil; 6 National Institute of Health and Care Research Moorfields Biomedical Research Center, Moorfields Eye Hospital, London, UK

**Keywords:** Proliferative diabetic retinopathy (PDR), Anti-VEGF therapy, Panretinal photocoagulation (PRP), Vitreous hemorrhage (VH), Tractional retinal detachment (TRD), Pars plana vitrectomy (PPV), Visual acuity, Diabetic macular edema (DME), Long-term outcomes, Meta-analysis

## Abstract

**Background:**

Studies diverge on the relevance of long-term protection of anti-VEGF against severe proliferative diabetic retinopathy (PDR) complications compared to pan-retinal photocoagulation (PRP). We aim to assess this dispute through a meta-analysis.

**Methods:**

We searched PubMed, Embase, and Cochrane databases until August 2024 for studies comparing anti-VEGF with PRP in PDR. Primary outcomes were long-term and short-term incidences of VH, TRD, and PPV—setting short-term follow-up up to 2 years and long-term follow-up over 5 years. Due to a lack of consistent data, TRD events were not stratified by clinical severity. We also evaluated diabetic macular edema (DME) rates and changes in best corrected visual acuity (BCVA) and central macular thickness (CMT). We used R to pool risk ratios (RR) and weighted mean differences with a random-effects model, and appraised evidence certainty using the GRADE tool. PROSPERO CRD42024577668.

**Results:**

We included eight studies with 12,812 eyes. Long-term data showed anti-VEGF reducing TRD (3.4% vs. 11.5%; RR 0.31, 95% CI 0.23–0.42; *p* = 0.001) with high certainty of evidence. However, PPV (7.8% vs. 9.4%; *p* = 0.116) and VH rates (11% vs. 18%; *p* = 0.38) did not differ, with moderate and low evidence certainty, respectively. In the short term, anti-VEGF demonstrated superiority in BCVA and CMT outcomes and reduced DME rates.

**Conclusions:**

Although anti-VEGF was associated with lower TRD rates in the long term, the absence of severity data and the lack of differences in PPV and VH raise questions about its clinical relevance. Long-term findings are limited to only two studies. Future research should stratify TRD by severity and include extended follow-up. In contrast, short-term outcomes consistently favored anti-VEGF for both visual and anatomical results.

**Supplementary Information:**

The online version contains supplementary material available at 10.1186/s40942-025-00687-0.

## Introduction

Historically, panretinal photocoagulation (PRP) has been the standard treatment for proliferative diabetic retinopathy (PDR) to prevent severe vision loss [1]. However, PRP is associated with a greater incidence of diabetic macular edema (DME) and loss of peripheral vision [1]. Alternatively, anti-VEGF therapy has been shown to improve vision, preserve peripheral visual field, and reduce the incidence of DME in short-term follow-up studies of 1–2 years, despite the need for frequent injections [2, 3]. 

Recent data suggests significant benefits of anti-VEGF therapy in preventing severe complications of PDR compared with PRP in the long run. Anti-VEGF had lower rates of vitreous hemorrhage (VH), tractional retinal detachment (TRD), and pars plana vitrectomy (PPV) over a 5-year period [4]. Notably, PROTOCOL S—a pivotal study comparing ranibizumab and PRP over the same period—reported considerably higher TRD rates in the PRP group. However, it did not underscore these findings. On the contrary, one of its main conclusions stated that serious PDR complications—such as macular TRD and severe vision loss—were negligible in both groups. These antagonist conclusions between long-term trials raise questions about the clinical relevance of anti-VEGF’s long-term protective effects against severe PDR complications [5]. 

To address this dispute over long-term outcomes and assess the clinical relevance of anti-VEGF therapy in preventing severe complications of PDR, we conducted a meta-analysis comparing anti-VEGF therapy with PRP over a five-year period.

## Methods

Our study was conducted and reported following the recommendations of the Cochrane Collaboration Handbook for Systematic Reviews of Interventions and the Preferred Reporting Items for Systematic Reviews and Meta-Analysis (PRISMA) Statement [6, 7]. The protocol has been registered prospectively with the International Prospective Register of Systematic Reviews (PROSPERO) under protocol number CRD42024577668.

### Data source and search strategy

We searched PubMed, Embase, and Cochrane databases up to August 2024. For the detailed search strategy, see the Supplemental Appendix. Authors T.R. and L.M. assessed each record using the Rayyan software. Conflicts were solved through consensus. Moreover, relevant studies were also sought by screening the references of eligible papers and systematic reviews.

### Eligibility criteria

There were no date or language restrictions. However, only studies published in English met the inclusion criteria. If non-English studies had been included, translations would have been obtained to facilitate consistent data extraction and quality assessment. Studies were considered eligible if they (1) were RCT and non-randomized observational studies; (2) directly compared intravitreal anti-VEGF monotherapy with PRP; (3) included patients diagnosed with PDR. The exclusion criteria were (1) conference abstracts, (2) duplicate publications, or (3) review articles.

### Endpoints

The main outcomes were long and short-term incidences of VH, TRD, and PPV. Due to inconsistent reporting, TRD events could not be stratified by clinical severity, such as the presence of macular involvement or the number of cases requiring surgery. We also assessed rates of DME, change in BCVA, and central macular thickness (CMT). Additional outcomes included dropout rates, safety outcomes, and adverse events. We settled short-term studies up to 2 years and long-term studies with follow-ups exceeding 5 years.

We settled these thresholds based on the chronic nature of diabetic retinopathy and the need to assess both early and sustained treatment effects, previous studies evaluating intervention´s short and long-term effects on diabetic retinopathy progression [8–10]and data availability.

Our study assessed individual outcomes rather than combining them into a composite endpoint, allowing for independent analysis and interpretation of each parameter’s clinical significance.

### Quality assessment

Two authors, T.R. and D.A., assessed the risk of bias in the included RCTs using the RoB-2 tool [11]. For non-randomized studies, the risk of bias was evaluated using the ROBINS-I tool [12]. Disagreements were resolved through consensus. We also assessed the certainty of evidence using the GRADE approach [6]. 

### Statistical analysis

We used R software’s metabin function from the meta package with the Mantel-Haenszel method (without continuity correction) to pool data for zero-event outcomes [13]. We calculated risk ratios (RRs) with 95% confidence intervals (CIs) for all binary endpoints and considered *p*-values below 0.05 statistically significant.

We assessed heterogeneity using Cochran’s Q and I² statistics, with *p* ≤ 0.10 indicating significant heterogeneity. We categorized I² values as follows: 0% for no, ≤ 25% for low, ≤ 50% for moderate, and > 50% for substantial heterogeneity. We performed all statistical analyses using R version 4.3.2 (R Foundation for Statistical Computing, Vienna, Austria).

We extracted values from graphs using WebPlotDigitizer [14] when data were not provided. If extraction failed, we contacted study authors for missing data. When standard deviations (SDs) were unavailable, we imputed them using the largest SD from the pooled analysis, following Cochrane Handbook recommendations. We collected individual study data in Excel (Microsoft Corporation, Redmond, WA, USA).

We conducted leave-one-out sensitivity analyses to ensure our results did not depend on any single study. In supplemental Table 1 (Supplemental Appendix), we collected and summarized other study characteristics, including demographics for intervention and control groups, study design, and funding sources.

In addition, we provided a summary table of the long-term studies’ main results and conclusions, contrasting their main findings with our analysis. (see *“Long-term studies Summary Table”* in the Supplemental Appendix).

## Results

### Study selection and characteristics

Our review yielded 929 results. After removing duplicates and screening based on title and abstract, we assessed 22 full-text articles for possible inclusion. Finally, eight studies fulfilled our inclusion criteria: seven RCTs [3, 5, 15–19] and one retrospective cohort study [4]. They comprised 12,812 patients, of whom 52.8% were male and 6,478 (50.06%) were allocated to the PRP group. Supplemental Fig. 1 provides details of the study selection.

The studies occurred across multiple countries, including the United States of America, the United Kingdom, Portugal, Syria, and Jordan. The mean age was 64.6 years. Total follow-up ranged from 1 year to 5 years, with 75% of the studies having a follow-up of 1 year, and 25% a follow-up of 5 years. All studies provided short-term follow-ups, and two studies provided long-term follow-ups. The mean number of injections was 6.13 (95% CI 4.54, 7.74; Supplemental Fig. 2). The agents most frequently used were ranibizumab and bevacizumab, each utilized in three studies. Aflibercept was used in only one study. Detailed characteristics of individual studies are presented in supplemental Table 1 (Supplemental Appendix).

### BCVA

Due to a lack of long-term data for BCVA, our analysis was limited to short-term results. Six studies [3, 5, 16–19] were included, with a mean follow-up of 1.16 years, ranging from 1 to 2 years. Five studies provided data for a 1-year follow-up [3, 16–19], while one provided a 2-year follow-up [5]. Anti-VEGF therapy showed significant improvement in BCVA compared with PRP, with a mean difference of 2.91 letters (95% CI 1.10, 4.7; *p* < 0.01; I^2^ = 0%; Supplemental Fig. 3A ). The leave-one-out sensitivity analysis highlighted the CLARITY trial [3] as an influential, since its exclusion changed the overall treatment effect, showing no difference between interventions (95% CI -0.38, 5.73; I^2^ = 15%; Supplemental Fig. 3B). Our GRADE assessment found high certainty of evidence for this outcome. For more information, refer to our GRADE assessment in the Supplemental Appendix.

### CMT and DME

Due to a lack of long-term data on CMT and DME rates, our analysis was limited to short-term results. For CMT, five studies^3,15,17–192−5^ were included with all presenting data for a 1-year follow-up. Anti-VEGF demonstrated a significant reduction in CMT compared with PRP, with a mean difference of -31.61 μm (95% CI -44.51 μm, -18.71 μm; *p* < 0.01; I^2^ = 50%; Supplemental Fig. 4A). In the leave-one-out sensitivity analysis, Shahraki 2022^19^ had an influential effect, as its exclusion reduced the heterogeneity to 0%. (Supplemental Fig. 4B).

For DME, five studies were included [3, 5, 15, 18, 19], with all presenting data for a 1-year follow-up. Anti-VEGF reduced the risk of DME by 63% compared to PRP (6.4% vs. 17.7%; RR 0.37; 95% CI 0.19–0.72; *p* = 0.003; I² = 37%; see Supplemental Fig. 5A).In the leave-one-out sensitivity analysis, Shahraki 2022^19^ had an influential effect, as its exclusion reduced the heterogeneity to 0%. Additionally, Protocol S [5] had an influential effect as its exclusion changed the overall effect, showing no differences between groups (Supplemental Fig. 5B).

Our GRADE assessment found high certainty of evidence for both outcomes. For more information, refer to the Supplemental Appendix.

### PPV rates

**Fig. 1:**
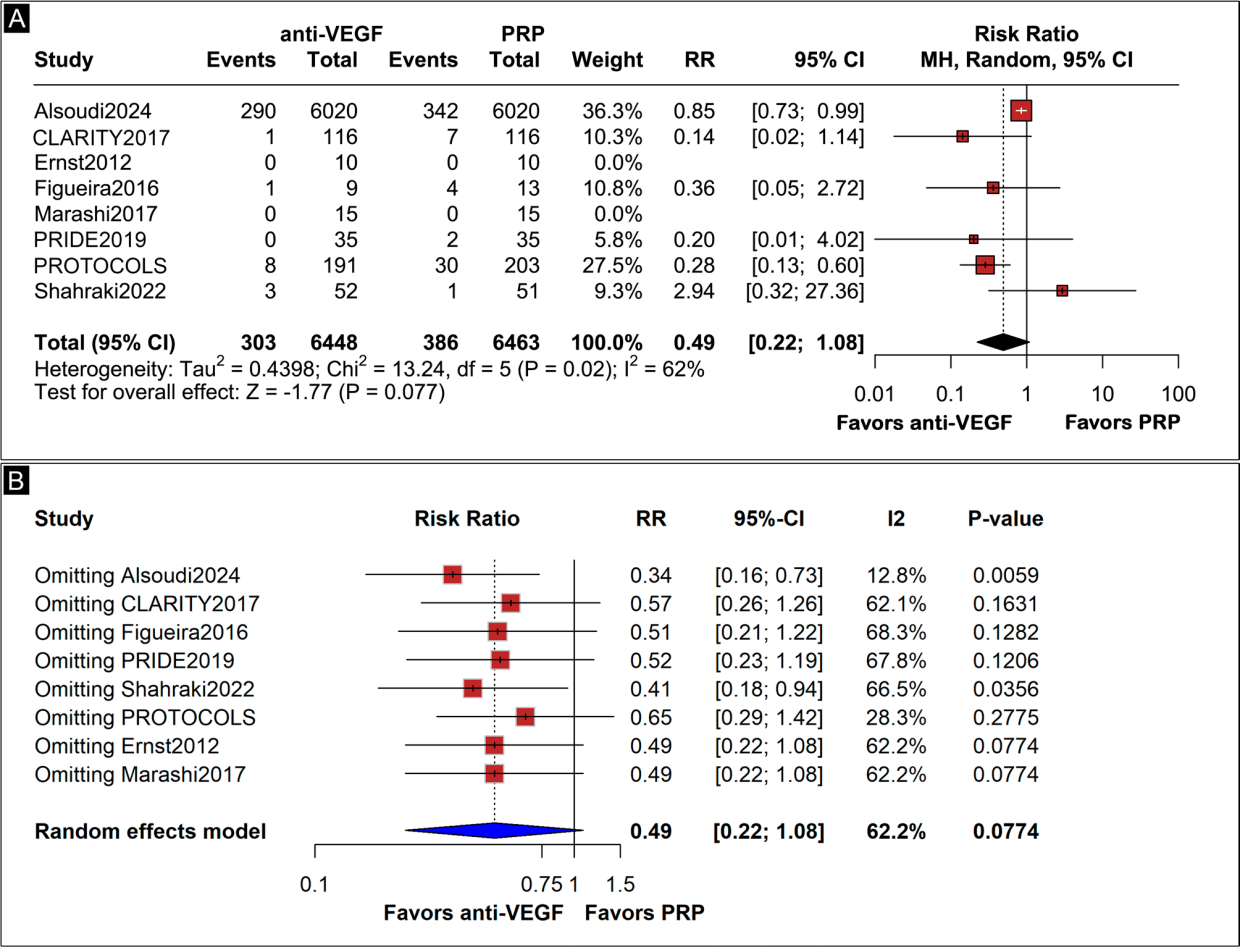
(**A**) Forest plot showing short-term pars plana vitrectomy (PPV) rates, with no significant difference between anti-VEGF and PRP groups (RR 0.49; 95% CI 0.22–1.08; *p* = 0.077; I² = 62%). Events: 303/6448 anti-VEGF patients vs. 386/6463 PRP patients. (**B**) Leave-one-out sensitivity analysis reveals that excluding Alsoudi et al. reduced heterogeneity to 13% and resulted in a significant effect favoring anti-VEGF (RR 0.34; 95% CI 0.16–0.73)

Six studies were analyzed for short-term PPV rates [3–5, 16, 18, 19] with a mean follow-up of 1.12 years, ranging from 1 to 2 years. Five studies provided data for a 1-year follow-up, while one study [5] provided data for a 2-year follow-up. There was no difference between groups, with anti-VEGF group presenting an incidence of 4.7% of the need for PPV versus 6% in the PRP groups (RR 0.49; 95% CI 0.22, 1.08; *p* = 0.077; I² = 62%; Fig. 1A). However, in the leave-one-out sensitivity analysis, excluding Alsoudi et al., [4] led to a statistical difference favoring anti-VEGF and dropping heterogeneity to 13%. Patients in the anti-VEGF group showed a 66% lower risk of requiring PPV when compared with the PRP group (RR, 0.34; 95% CI 0.16, 0.73; *p* = 0,006; I^2^ = 13%; Fig. 1B). We found a high certainty of evidence for this outcome upon GRADE assessment.

**Fig. 2:**
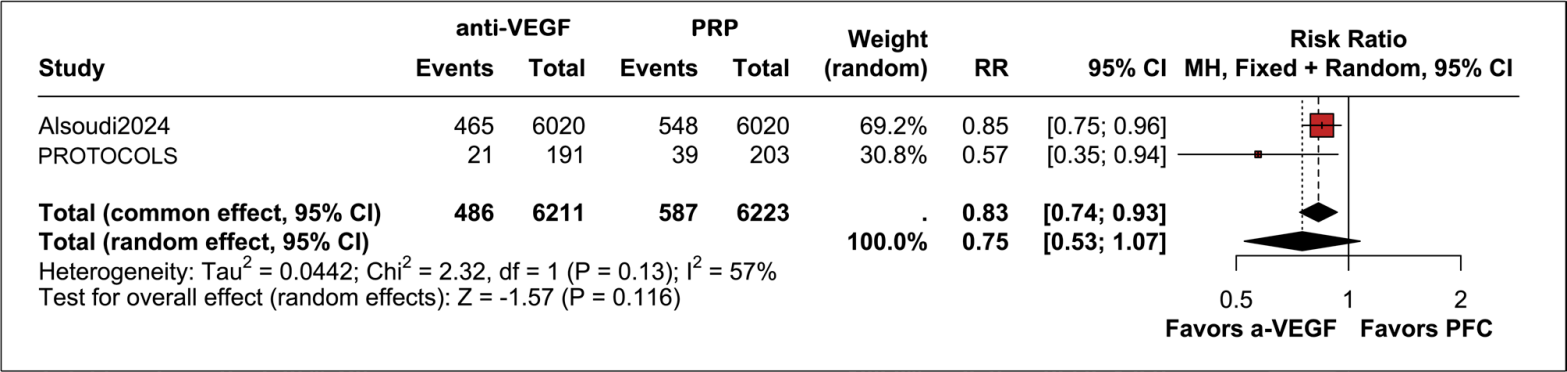
Forest plot showing long-term PPV rates, with no significant difference between anti-VEGF and PRP groups (RR 0.75; 95% CI 0.53–1.07; *p* = 0.116; I² = 57%). Events: 486/6211 anti-VEGF patients vs. 587/6223 PRP patients. Heterogeneity was moderate, with results derived from two studies

We included two studies for long-term outcomes in PPV rates, both with a 5-year follow-up [4, 5]. There was no difference between anti-VEGF therapy and PRP in terms of PPV, with 7.8% of patients in the anti-VEGF group needing surgery versus 9.4% in the PRP group (RR 0.75; 95% CI 0.53, 1.07; *p* = 0.116; I²=57%; Fig. 2), with a moderate certainty of evidence upon GRADE assessment. The main reason for downgrading was the moderate heterogeneity presented in this outcome (see the GRADE assessment in the *Supplemental Appendix)*.

### VH rates

**Fig. 3:**
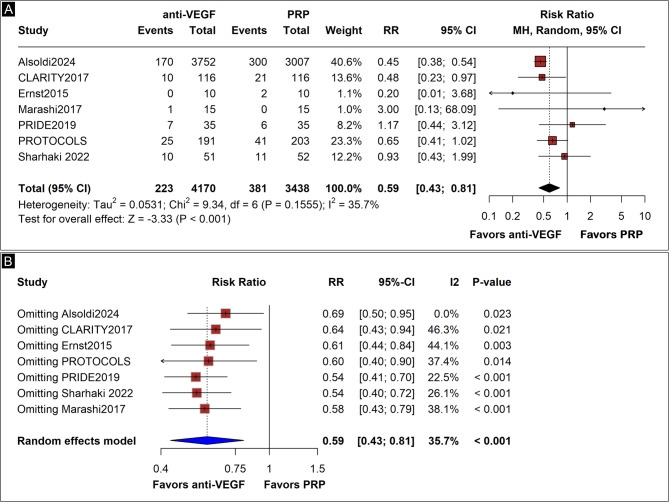
(**A**) Forest plot showing short-term vitreous hemorrhage (VH) rates, with significantly lower rates in the anti-VEGF group compared with PRP (RR 0.59; 95% CI 0.43–0.81; *p* < 0.001; I² = 36%).Events: 223/4170 anti-VEGF patients vs. 381/3438 PRP patients. (**B**) Leave-one-out sensitivity analysis confirms the robustness of the pooled effect; excluding Alsoudi et al. reduced heterogeneity to 0%

We included seven studies in the short-term analysis of VH rates [3–5, 15, 17–19]. all providing data of a 1-year follow-up. Anti-VEGF therapy reduced the risk of VH by 41% compared to PRP (5.3% vs. 11.1%; RR 0.59; 95% CI 0.43–0.81; *p* = 0.001; I² = 36%; Fig. 3A). In the leave-one-out sensitivity analysis, removing Alsoudi 2024^4^ reduced the heterogeneity to 0% (Fig. 3B). We found a high certainty of evidence for this outcome upon GRADE assessment.

**Fig. 4:**
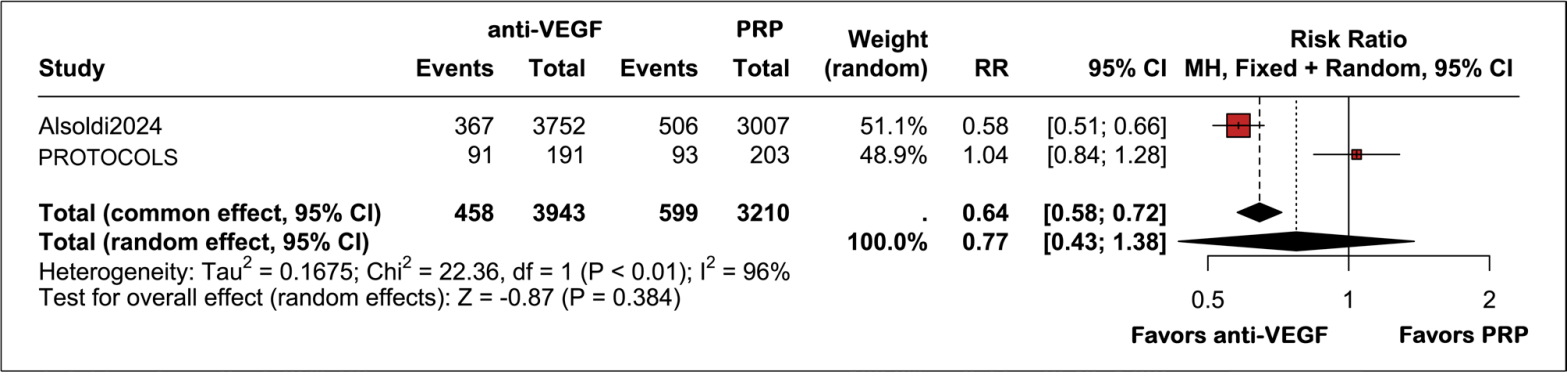
Forest plot showing long-term VH rates, with no significant difference between anti-VEGF and PRP groups (RR 0.77; 95% CI 0.43–1.38; *p* = 0.384; I² = 96%). Events: 458/3943 anti-VEGF patients vs. 599/3210 PRP patients. High heterogeneity indicates substantial variability between the studies

We included two studies in the analysis of long-term outcomes for VH rates, all presenting data at 5 years [4, 5]. We found no difference between groups with an 11% incidence of VH in the anti-VEGF group versus an 18% incidence of this outcome in the PRP group (RR 0.77; 95% CI 0.43, 1.38; *p* = 0.38; I² = 96%; Fig. 4). Our GRADE assessment found a low certainty of evidence for this outcome (see Supplemental Appendix). The main reasons for downgrading were high heterogeneity and imprecision.

### TRD rates

We included seven studies for the short-term TRD outcomes, with a mean follow-up duration of 1.14 years, ranging from 1 to 2 years [3–5, 15, 18, 19]. Six studies provided data for a 1-year follow-up, while one study [5] provided data for a 2-year follow-up. Due to the low event rate of TRD, our statistical analysis was limited. In the anti-VEGF group, 2% of the patients developed TRD (90 eyes out of 4,114), and 6% developed TRD in the PRP group (236 eyes out of 3,422). However, this numerical advantage favoring anti-VEGF did not reach statistical significance (RR 0.63; 95% CI 0.21, 1.90; *p* = 0.41; I^2^ = 82%; Supplemental Fig. 6). Our GRADE assessment found a very low certainty of evidence for this outcome. The main reasons for downgrading were high heterogeneity, which was not explained by sensitivity analysis, and high imprecision due to wide CIs.

**Fig. 5:**
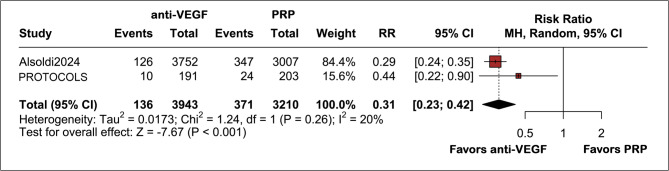
Forest plot showing long-term tractional retinal detachment (TRD) rates, with significantly lower rates in the anti-VEGF group compared to PRP (RR 0.31; 95% CI 0.23–0.42; *p* < 0.001; I² = 20%). TRD was analyzed as a binary outcome, regardless of clinical severity (i.e., including all events regardless of macular involvement or surgical need). Events: 136/3943 anti-VEGF patients vs. 371/3210 PRP patients. Low heterogeneity indicates consistent results across studies

Two studies were included for the long-term TRD outcome, with both of these studies presenting data at 5 years [4, 5]. Anti-VEGF therapy reduced the risk of TRD by 69% compared to PRP (3.4% vs. 11.5%; RR 0.31; 95% CI 0.23–0.42; *p* < 0.001; I² = 20%; Fig. 5) with low heterogeneity across the studies. Our GRADE assessment rated the evidence as having high certainty due to precise findings with minimal heterogeneity. Additionally, the significant effect size enhances the reliability of these results (see Supplemental Appendix). However, it is essential to note that TRD events were not stratified by clinical severity, such as macular involvement or the need for surgical intervention.

## Discussion

This meta-analysis of eight studies examining 12,812 eyes compared anti-VEGF monotherapy with PRP in the short and long term. Short-term results showed that anti-VEGF outperformed PRP in terms of changes in BCVA and CMT, as well as in rates of DME and VH. Overall, there was no significant difference in PPV rates, but a subanalysis including only RCTs showed benefits for the anti-VEGF group. Concerning short-term TRD rates, most studies reported zero events, which hindered our statistical analysis. Even so, we found no difference between groups. In the long term, both groups had similar PPV and VH rates, although we found a considerably higher risk of TRD in the PRP group.

Our short-term findings on changes in BCVA and CMT, as well as on rates of DME and VH, align with the existing literature, showing the superiority of anti-VEGF over PRP for treating PDR [3, 5, 18]. Concerning PPV rates, as we applied the sensitivity analysis, removing the only observational study in the pooled data [4], we also observed this trend of favoring anti-VEGF. The inclusion of Alsoudi and colleagues likely changed the overall results due to their observational design, which had limited control over confounders and resulted in smaller effect sizes. Combined with its high weight in the pooled data, this reduced the overall positive impact of anti-VEGF, leading to no significant difference between groups.

For long-term outcomes, TRD occurred in 11.5% of PRP patients versus 3.4% with anti-VEGF—a 69% lower risk with anti-VEGF, supported by high-certainty evidence. These findings are consistent with the recent publication by Alsoudi and colleagues [4], which also reported fewer instances of TRD in patients with anti-VEGF. Notably, our meta-analysis indicated that Protocol S, over a 5-year follow-up, also showed a protective effect of anti-VEGF for TRD compared with PRP in its data. However, this was not emphasized in the original study results. They only displayed the numerical values in their supplemental material and did not perform any measure of association [5]. ( see more in *Summary Table on Long-term Studies* in the Supplemental Appendix)

Our long-term PPV rates did not differ between groups, which conflicted with PROTOCOL S and Alsoudi, et al. [4, 5] Nonetheless, the results favoring anti-VEGF were weak in both studies [4], with a moderate to small effect size and a CI near the null. The contrasting findings in our meta-analysis between TRD and PPV rates challenge the clinical validity of anti-VEGF’s superiority over PRP against TRD, conflicting with the main conclusions of Alsoudi and colleagues [4]. Notably, in contrast to Alsoudi’s work, PROTOCOL S did not emphasize anti-VEGF protection against severe complications of PDR. They considered serious complications of PDR to be negligible in both groups (for a detailed comparison, refer to our *Long-Term Studies Summary Table* in the Supplemental Appendix). A closer review of Protocol S [5] showed that although the PRP group had a higher TRD rate, most cases were mild—only 6 of 34 cases required surgery (2 in the anti-VEGF group vs. 4 in PRP) in a cohort of 394 patients. Moreover, vision outcomes were similar between groups despite the higher TRD incidence, reinforcing our assertion that the protective effect of anti-VEGF may lack clinical significance.

Previous systematic reviews, such as those by Macaron et al. (2025) and Simmonds et al. (2024), provided a systematic analysis, but with important distinctions. Macaron et al. focused on short-term functional outcomes, such as BCVA and neovascular regression, while also assessing combination therapy (anti-VEGF plus PRP). In contrast, Simmonds et al. conducted a network meta-analysis comparing different anti-VEGF agents, primarily evaluating changes in vision over 1 to 2 years. Neither review aimed to assess long-term outcomes beyond two years. Our study is novel in providing a pooled assessment of long-term complications. Additionally, it reinforces the importance of stratifying TRD severity to facilitate better clinical decision-making.

Our study has significant limitations. Rates of TRD alone have limited relevance. A more appropriate endpoint would be TRD requiring surgery or involving the macula. Most of the studies did not report this event in that manner. Protocol S [5] was the only long-term trial that provided data on patients who needed surgery, and none provided data on macular TRD rates. Another limitation is limited long-term data [4, 5]., which limits generality and hinders the ability to analyze critical long-term outcomes such as BCVA, quality of life, visual field loss, the incidence of DME, and whether different anti-VEGF types would change the overall outcomes. Additionally, a significant challenge in anti-VEGF therapy is clinical feasibility, which has a high treatment burden and economic implications, which we could only assess in the short term. Data show that, in the long run, nearly half of the patients on anti-VEGF tend to discontinue their treatment [20]. This becomes more complex because evidence suggests that patients with PDR who discontinued their anti-VEGF treatment experienced significantly worse outcomes than those who underwent laser treatment and then dropped out [21]. Furthermore, we were unable to assess the different anti-VEGFs’ efficacy due to limited data.

Previous systematic reviews have provided valuable analyses with essential distinctions. Simmonds et al. conducted a robust meta-analysis incorporating partial individual patient data (IPD) from approximately 33% of the pooled sample [22]. IPD enables more consistent and flexible modeling through the reanalysis of individual-level data. Their findings supported the short-term benefits of anti-VEGF in reducing DME, which aligns with our results; however, they were unable to assess long-term outcomes due to the limited data available. Also, even with partial IPD access, they could not stratify TRD by severity, further underscoring a critical gap in the literature and the need for future studies to report this information systematically. Macaron et al. evaluated anti-VEGF, PRP, and combination therapy, but their analysis was similarly limited to short- and mid-term outcomes (up to two years) [23]. 

Our meta-analysis is the first to evaluate the long-term efficacy of anti-VEGF therapy compared to PRP in PDR. Anti-VEGF therapy showed clear short-term benefits, including lower rates of DME, VH, and PPV, along with modest improvement in visual acuity. Long-term results showed significantly lower TRD rates with anti-VEGF therapy, but similar PPV rates and inconsistent VH results. Our analysis also found that most studies did not stratify TRD by severity; those that did, such as PROTOCOL S, revealed that most TRDs were mild and non-surgical. These findings suggest that the clinical significance of anti-VEGF protection against severe PDR complications may be limited. Given that only two studies contributed to the long-term analysis, further randomized trials with detailed TRD stratification are needed to clarify the long-term comparative efficacy of anti-VEGF therapy versus PRP.

## Electronic supplementary material

Below is the link to the electronic supplementary material.

Supplementary Material 1

## Data Availability

No datasets were generated or analysed during the current study.
