# Supplementary material for: Meta-analysis: long/short-term efficacy of anti-VEGF vs. panretinal photocoagulation in preventing severe complications in proliferative diabetic retinopathy
Source: Int J Retina Vitreous. 2025 Jul 9;11:76. doi: 10.1186/s40942-025-00687-0 (PMC12239425; doi:10.1186/s40942-025-00687-0)
Supplement: Supplementary file 1 — Supplementary Material 1 [file 40942_2025_687_MOESM1_ESM.docx]

**SUPPLEMENTAL APPENDIX**

**Meta-analysis: Long/short-term efficacy of anti-VEGF vs panretinal photocoagulation in preventing severe complications in proliferative diabetic retinopathy**

**Tiago N O Rassi, MD^1,2^; Lucas M Barbosa^3^; Dillan C Amaral^4^; Ricardo N Louzada, MD, PhD^4^; Helvécio N F Filho^5^; Guilherme N Marques^4^; Breno C Vieira^4^; Sobha Sivaprasad MD, PhD^6^; Mauricio Maia, MD, PhD^2^;**

**Table of Contents**

**Supplemental Methods 1. PRISMA 2020 Main Checklist – Page 2-3**

**Supplemental Methods 2. PRISMA Abstract Checklist – Page 4**

**Supplemental Methods 3. Details of the Search Strategy – Page 5**

**Supplemental Methods 4. Full Text Reviewed Studies – Page 5**

**Supplemental Methods 5. Risk of Bias – Page 5-7**

**Supplemental Methods 6. Dropout rates, serious ocular events, and Systemic Adverse Events – Page 7-8**

**Supplemental Methods 7. GRADE assessment – Page 9**

**Supplemental Figures – Page 10-14**

**Supplemental Table 1- Page 15-16**

**Summary Table on Long-Term Studies- Page 17**

**Supplemental Methods 1.** PRISMA 2020 Main

| **Topic** | **No** | **Item** | **Location where item is reported** |
| --- | --- | --- | --- |
| **TITLE** |  |  |  |
| **Title** | 1 | Identify the report as a systematic review. | Pg. 1 at MS |
| **ABSTRACT** |  |  |  |
| **Abstract** | 2 | See the PRISMA 2020 for Abstracts checklist | Pg. 4 at sup |
| **INTRODUCTION** |  |  |  |
| **Rationale** | 3 | Describe the rationale for the review in the context of existing knowledge. | Pg. 4 at MS |
| **Objectives** | 4 | Provide an explicit statement of the objective(s) or question(s) the review addresses. | Pg. 4 at MS |
| **METHODS** |  |  |  |
| **Eligibility criteria** | 5 | Specify the inclusion and exclusion criteria for the review and how studies were grouped for the syntheses. | Pg 5 at MS/pg 5 supp appendix |
| **Information sources** | 6 | Specify all databases, registers, websites, organizations, reference lists and other sources searched or consulted to identify studies. Specify the date when each source was last searched or consulted. | Pg. 5 at MS |
| **Search strategy** | 7 | Present the full search strategies for all databases, registers, and websites, including any filters and limits used. | Pg 5 supp appendix |
| **Selection process** | 8 | Specify the methods used to decide whether a study met the inclusion criteria of the review, including how many reviewers screened each record and each report retrieved, whether they worked independently, and if applicable, details of automation tools used in the process. | Pg. 5 at MS |
| **Data collection process** | 9 | Specify the methods used to collect data from reports, including how many reviewers collected data from each report, whether they worked independently, any processes for obtaining or confirming data from study investigators, and if applicable, details of automation tools used in the process. | Pg. 7 at MS |
| **Data items** | 10a | List and define all outcomes for which data were sought. Specify whether all results that were compatible with each outcome domain in each study were sought (e.g., for all measures, time points, analyses), and if not, the methods used to decide which results to collect. | Pg. 6-7 at MS |
|  | 10b | List and define all other variables for which data were sought (e.g., participant and intervention characteristics, funding sources). Describe any assumptions made about any missing or unclear information. | Pg. 6-7 at MS |
| **Study risk of bias assessment** | 11 | Specify the methods used to assess risk of bias in the included studies, including details of the tool(s) used, how many reviewers assessed each study and whether they worked independently, and if applicable, details of automation tools used in the process. | Pg. 6 at MS |
| **Effect measures** | 12 | Specify for each outcome the effect measure(s) (e.g., risk ratio, mean difference) used in the synthesis or presentation of results. | Pg 7 at MS |
| **Synthesis methods** | 13a | Describe the processes used to decide which studies were eligible for each synthesis (e.g., tabulating the study intervention characteristics and comparing against the planned groups for each synthesis (item 5)). | Table 1, Pg 7 at MS |
|  | 13b | Describe any methods required to prepare the data for presentation or synthesis, such as handling of missing summary statistics, or data conversions. | Pg. 7 at MS |
|  | 13c | Describe any methods used to tabulate or visually display results of individual studies and syntheses. | Table 1 |
|  | 13d | Describe any methods used to synthesize results and provide a rationale for the choice(s). If meta-analysis was performed, describe the model(s), method(s) to identify the presence and extent of statistical heterogeneity, and software package(s) used. | Pg. 6-7 at MS |
|  | 13e | Describe any methods used to explore possible causes of heterogeneity among study results (e.g., subgroup analysis, meta-regression). | Pg. 7 at MS |
|  | 13f | Describe any sensitivity analyses conducted to assess robustness of the synthesized results. | Pg. 7 at MS |
| **Reporting bias assessment** | 14 | Describe any methods used to assess risk of bias due to missing results in a synthesis (arising from reporting biases). | NA |
| **Certainty assessment** | 15 | Describe any methods used to assess certainty (or confidence) in the body of evidence for an outcome. | Pg. 6 at MS |
| **RESULTS** |  |  |  |
| **Study selection** | 16a | Describe the results of the search and selection process, from the number of records identified in the search to the number of studies included in the review, ideally using a flow diagram. | Pg 7-8 of the MS and Figure 1 |
|  | 16b | Cite studies that might appear to meet the inclusion criteria, but which were excluded, and explain why they were excluded. | Pg 5 Supp app |
| **Study characteristics** | 17 | Cite each included study and present its characteristics. | Table 1 |
| **Risk of bias in studies** | 18 | Present assessments of risk of bias for each included study. | Pg 5-7 Supp app |
| **Results of individual studies** | 19 | For all outcomes, present, for each study: (a) summary statistics for each group (where appropriate) and (b) an effect estimates and its precision (e.g., confidence/credible interval), ideally using structured tables or plots. | Fig 1-5 and Supplemental Figures |
| **Results of syntheses** | 20a | For each synthesis, briefly summarize the characteristics and risk of bias among contributing studies. | Pg 7-11 at MS and pg 5-7 Supp app |
|  | 20b | Present results of all statistical syntheses conducted. If meta-analysis was done, present for each the summary estimate and its precision (e.g., confidence/credible interval) and measures of statistical heterogeneity. If comparing groups, describe the direction of the effect. | Pg. 7-11 at MS |
|  | 20c | Present results of all investigations of possible causes of heterogeneity among study results. | Pg. 8-13 at MS |
|  | 20d | Present results of all sensitivity analyses conducted to assess the robustness of the synthesized results. | Pg. 7-11 at MS, 1-5 and Supplemental Figures |
| **Reporting biases** | 21 | Present assessments of risk of bias due to missing results (arising from reporting biases) for each synthesis assessed. | NA |
| **Certainty of evidence** | 22 | Present assessments of certainty (or confidence) in the body of evidence for each outcome assessed. | Pg. 7-11 ate MS and pg 9 of Supp app |
| **DISCUSSION** |  |  |  |
| **Discussion** | 23a | Provide a general interpretation of the results in the context of other evidence. | Pg. 12-15 at MS |
|  | 23b | Discuss any limitations of the evidence included in the review. | Pg. 12-15 at MS |
|  | 23c | Discuss any limitations of the review processes used. | Pg. 12-15 at MS |
|  | 23d | Discuss implications of the results for practice, policy, and future research. | Pg. 12-15 at MS |
| **OTHER INFORMATION** |  |  |  |
| **Registration and protocol** | 24a | Provide registration information for the review, including register name and registration number, or state that the review was not registered. | PROSPERO; CRD42024577668 |
|  | 24b | Indicate where the review protocol can be accessed, or state that a protocol was not prepared. | <https://www.crd.york.ac.uk/prospero/display_record.php?RecordID=577668> |
|  | 24c | Describe and explain any amendments to information provided at registration or in the protocol. | We added GRADE assessment to test the certainty of our evidence. |
| **Support** | 25 | Describe sources of financial or non-financial support for the review, and the role of the funders or sponsors in the review. | None |
| **Competing interests** | 26 | Declare any competing interests of review authors. | NA |
| **Availability of data, code and other materials** | 27 | Report which of the following are publicly available and where they can be found template data collection forms; data extracted from included studies; data used for all analyses; analytic code; any other materials used in the review. | NA |

^Abbreviations: MS, manuscript; NA, not available; pg., page; sup App., supplemental appendix.^

**Supplemental Methods 2.** PRISMA Abstract Checklist

| **Topic** | **No.** | **Item** | **Reported?** |
| --- | --- | --- | --- |
| **TITLE** |  |  |  |
| **Title** | 1 | Identify the report as a systematic review. | Yes |
| **BACKGROUND** |  |  |  |
| **Objectives** | 2 | Provide an explicit statement of the main objective(s) or question(s) the review addresses. | Yes |
| **METHODS** |  |  |  |
| **Eligibility criteria** | 3 | Specify the inclusion and exclusion criteria for the review. | Yes |
| **Information sources** | 4 | Specify the information sources (e.g., databases, registers) used to identify studies and the date when each was last searched. | Yes |
| **Risk of bias** | 5 | Specify the methods used to assess risk of bias in the included studies. | No |
| **Synthesis of results** | 6 | Specify the methods used to present and synthesize results. | Yes |
| **RESULTS** |  |  |  |
| **Included studies** | 7 | Give the total number of included studies and participants and summarize relevant characteristics of studies. | Yes |
| **Synthesis of results** | 8 | Present results for main outcomes, preferably indicating the number of included studies and participants for each. If meta-analysis was done, report the summary estimate and confidence/credible interval. If comparing groups, indicate the direction of the effect (i.e., which group is favored). | Yes |
| **DISCUSSION** |  |  |  |
| **Limitations of evidence** | 9 | Provide a brief summary of the limitations of the evidence included in the review (e.g., study risk of bias, inconsistency, and imprecision). | No |
| **Interpretation** | 10 | Provide a general interpretation of the results and important implications. | Yes |
| **OTHER** |  |  |  |
| **Funding** | 11 | Specify the primary source of funding for the review. | No |
| **Registration** | 12 | Provide the register name and registration number. | Yes |

**Supplemental Methods 3. Details of the Search Strategy**

**Pubmed and Cochrane**

("Anti-VEGF" OR bevacizumab OR ranibizumab OR aflibercept OR faricimab) AND (PDR OR "proliferative diabetic retinopathy" OR "high risk diabetic retinopathy" OR "high-risk diabetic retinopathy") AND (laser OR "panretinal photocoagulation" OR PRP OR "panretinal photocoag*")

**Embase**

('Anti-VEGF'/exp OR bevacizumab/exp OR ranibizumab/exp OR aflibercept/exp OR faricimab/exp) AND ('proliferative diabetic retinopathy'/exp OR PDR OR 'high risk diabetic retinopathy' OR 'high-risk diabetic retinopathy') AND (laser/exp OR 'panretinal photocoagulation'/exp OR PRP OR 'panretinal photocoag*')

**Supplemental Methods 4. Full Text Reviewed Studies**

**Supplemental Methods 5. Risk of bias and quality assessment**

Using the ROBINS-I tool, the Alssoudi study presented moderate confounding since not all potential confounders were controlled despite using propensity score. The risk of participant selection and classification of interventions was low, indicating a well-defined and representative selection of participants and identified interventions. However, the study presented moderate risk related to missing data and the selection of reported results, mainly due to the lack of adjustments for multiple comparisons in the statistical analysis, which could influence the results favorably. Measuring outcomes and deviations from planned interventions presented a low risk, reflecting reasonable methodological control.

**Risk of bias summary for non-randomized studies (ROBINS - I)**

In the ROB-2 assessment, CLARITY showed a low risk of bias in the randomization and outcome measurement processes, although there were concerns regarding blinding and handling of missing data. Similarly, Figueira demonstrated a low risk in all domains, reflecting a robust methodology. In contrast, Ernst faced a high risk of bias due to significant loss to follow-up, which could compromise the reliability of the results. The PROTOCOLS, conducted in 2018, presented some concerns related to loss of follow-up. Marashi's study showed an overall low risk but with some reservations due to flexibility in the interventions, which could impact adherence to the original protocol. Similarly, Pride was conducted with a low risk of bias in most domains, although it faced missing data due to discontinuation and loss of follow-up. Finally, the 2022 Shahraki study demonstrated low risk in several domains but faced challenges managing missing data and the potential for selective reporting. Also, it made a per-protocol analysis, which may have hindered the randomization process.

**Risk of bias summary for randomized studies (ROB2)**

**Supplemental Methods 6. Dropout rates, serious ocular events, and Systemic Adverse Events**

**Dropout rates**

We included six studies in this outcome, with a mean follow-up of 1.16, ranging from 1 to 2 years. All studies provided outcomes at 1 year, except for PROTOCOL S^5^, which provided data for a 2-year follow-up. The dropout rate in the anti-VEGF group was 11.7% (52 eyes) and in the PRP group was 11.7% (57 eyes) with no difference between both groups (RR 1.09; 95 % CI 0.57, 2.07; p = 0.79; I^2^=36%; Supplemental Figure 2). Our leave-one-out sensitivity analysis found the CLARITY trial to be a source of heterogeneity (RR 1.18; 95% CI 0.75, 1.83; I^2^=0%). We could not assess long-term dropout rates due to a lack of data.


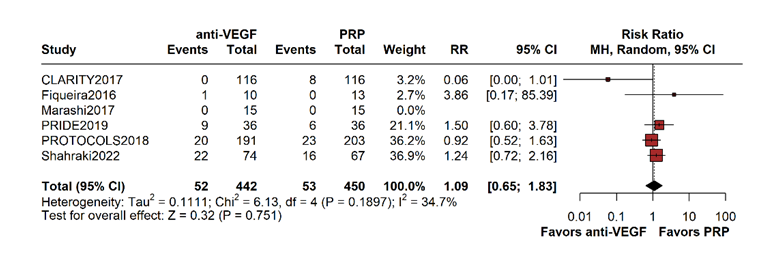


Forest plot comparing dropout rates between the anti-VEGF and PRP groups across the included studies. The risk ratio (RR) and 95% confidence intervals (CIs) were calculated using a Mantel-Haenszel random-effects model. Individual study estimates are represented by red squares, with the size of each square proportional to the study weight. The horizontal lines denote the 95% CIs, and the pooled estimate is represented by the black diamond at the bottom of the plot. A risk ratio greater than 1 favors PRP, whereas a value below 1 favors anti-VEGF. The overall pooled RR was 1.09 (95% CI: 0.65–1.83, p = 0.751), indicating no significant difference in dropout rates between the two treatment groups. Heterogeneity was low to moderate (I² = 34.7%), suggesting some variation across studies but without substantial inconsistency.

**Serious Ocular Adverse Events**

There were 27 cases of neovascular glaucoma/iris neovascularization, with 14 in the anti-VEGF group and 13 in the PRP group. Only Protocol S^5^ reported cases of retinal detachment—12 cases in anti-VEGF and 30 in PRP—with the type unspecified. Additionally, the anti-VEGF group had one reported case of endophthalmitis (PROTOCOL S) and one case of a retinal tear (CLARITY TRIAL).

**Systemic Adverse Events**

As for thromboembolic events, we found an incidence of 4% in the anti-VEGF group versus 3% in the PRP group with no statistical difference (RR 1.46; 95% CI 0.66, 3.24; p = 0.35; I^2^=0%; Supplemental Figure 6).

**Supplemental Methods 7. GRADE assessment**

RCT denotes randomized controlled trial. PRP denotes Panretinal Photocoagulation. PPV denotes Pars Plana Vitrectomy. VH denotes Vitreous hemorrhage. TRD denotes Tractional Retinal Detachment. BCVA denotes best corrected visual acuity. CMT denotes Central Macular Thickness. DME denotes diabetic macular edema.

a- Despite Ernst et al. being classified as a high risk of bias, we did not consider that it would harm the results due to its low weight in the pooled results, and its removal did not change the overall size effects and heterogeneity.

b- Moderate heterogeneity was justified by the presence of Sharaki in the analysis, as shown in our leave-one-out analysis. Sharaki used a per-protocol analysis, which could have biased the results.

c-While the presence of Ernst may increase the risk of bias, we did not consider downgrading due to the fact that this study did not have enough power to change the overall results due to its limited sample sizes.

d-Many studies reported no events hindering the power of our analysis. The leave-one-out sensitivity analysis could not explain high heterogeneity.

e-The study presented with wide confidence intervals.

**Supplemental Figures**

**Supplemental Figure 1. Study Selection**


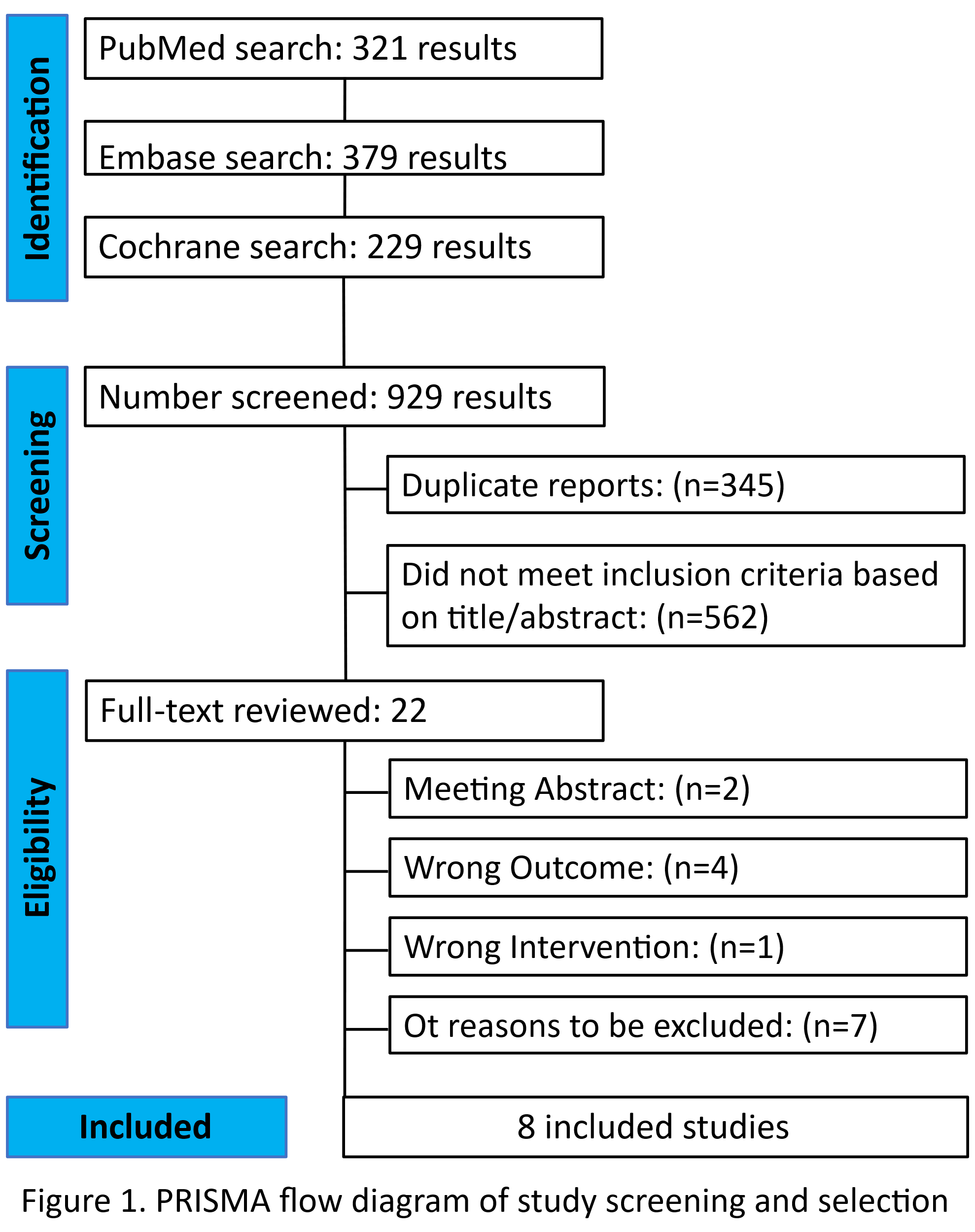


Supplemental Figure 1. PRISMA flow diagram illustrating the study selection process. A total of 929 records were identified through database searches, screened for eligibility, and filtered down to eight included studies after removing duplicates and applying inclusion criteria.

**Supplemental Figure 2. The mean number of injections in the anti-VEGF studies**

**
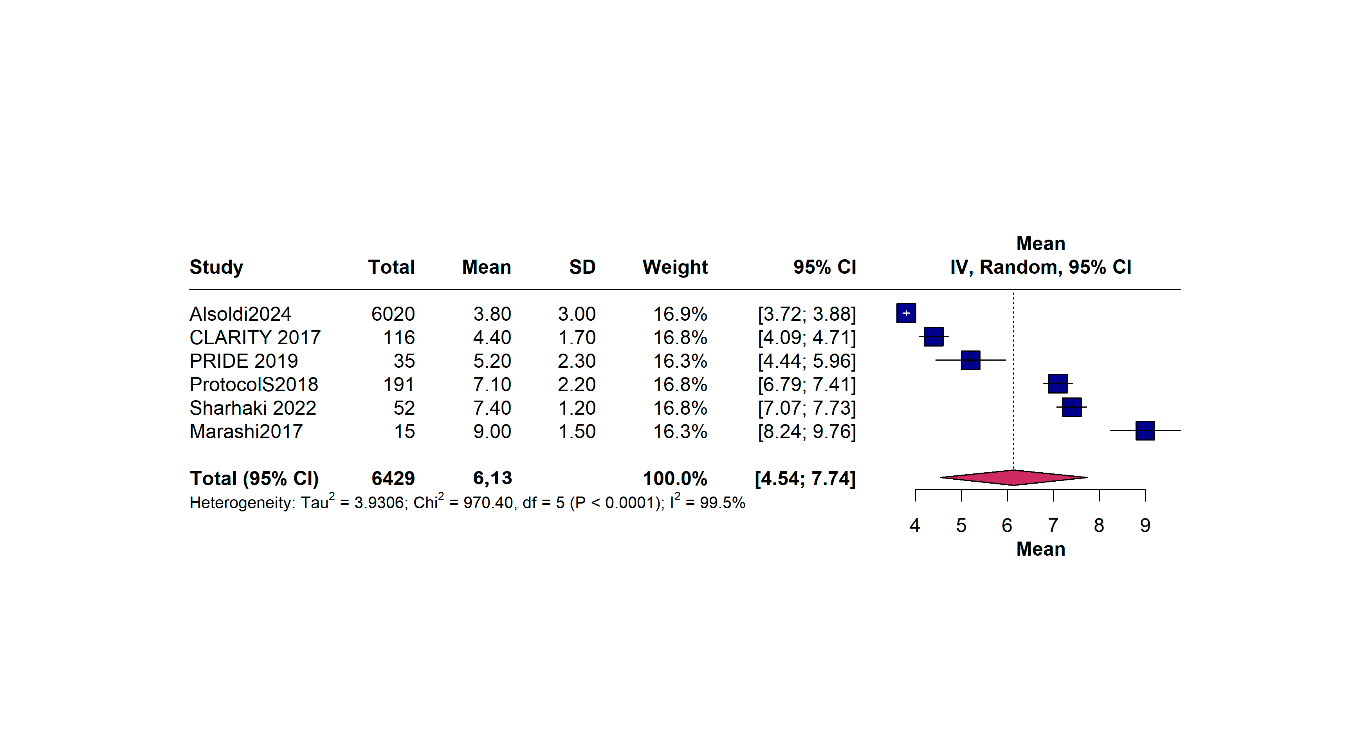
**

Supplemental figure 2. Forest plot displaying the mean number of intravitreal anti-VEGF injections administered over the follow-up period in the included studies. The analysis follows a random-effects model, with individual study estimates represented by blue squares, the size of which reflects the study weight. The horizontal lines indicate the 95% confidence intervals (CIs) for each study. The pooled mean estimate, calculated using the inverse variance (IV) method, is represented by the red diamond, with its width denoting the 95% CI. High heterogeneity was observed (I² = 99.5%), indicating substantial variability among the included studies.

**Supplemental Figure 3. Best Corrected Visual Acuity**


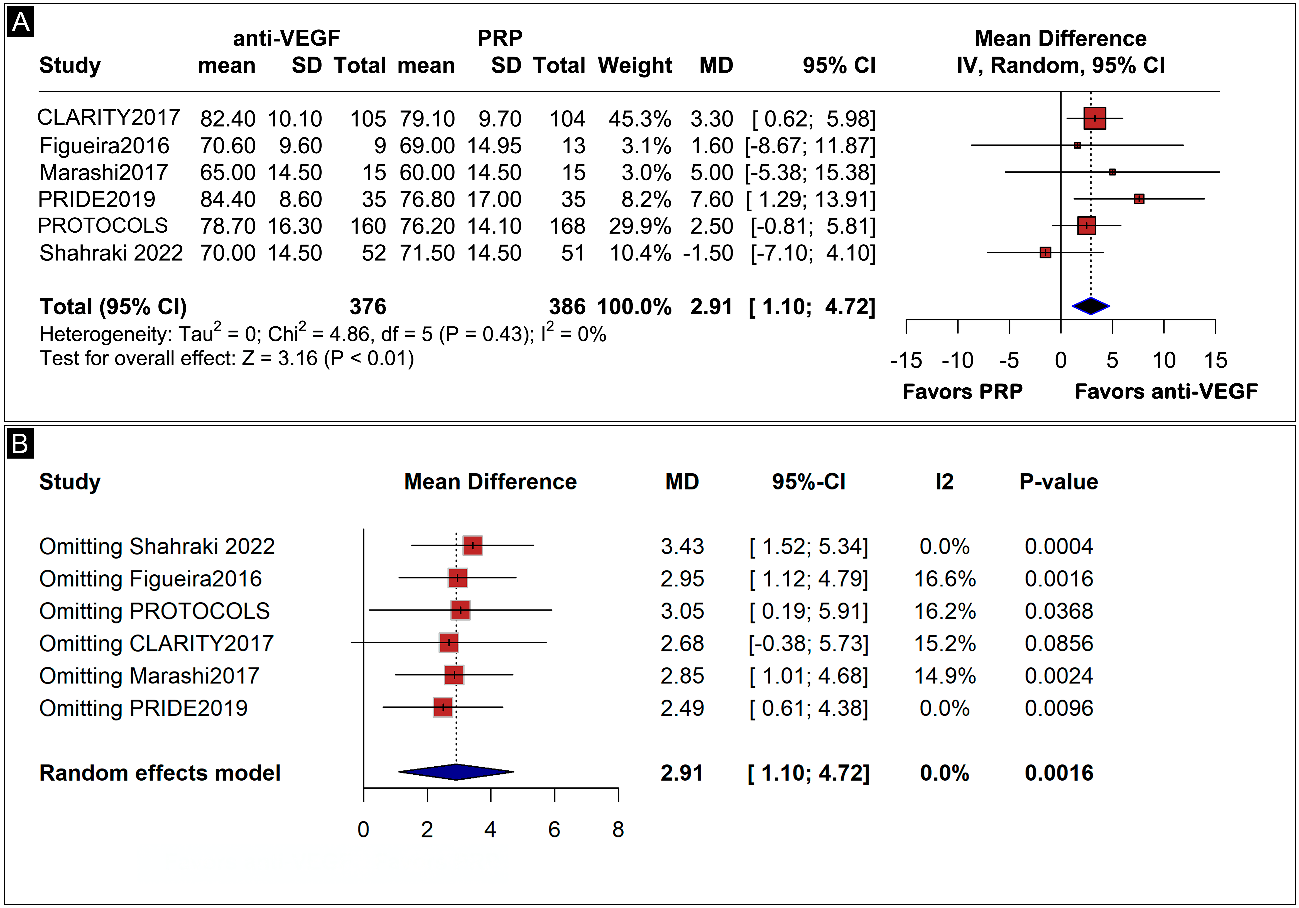


Supplemental Figure 3. (A) Forest plot comparing the mean difference in best-corrected visual acuity (BCVA) between anti-VEGF and PRP groups, showing significant improvement favoring anti-VEGF (MD 2.91; 95% CI 1.10, 4.72; p < 0.01; I² = 0%). (B) Leave-one-out sensitivity analysis showing the CLARITY trial as influential, with its exclusion significantly altering the overall effect size, demonstrating its impact on the pooled results.

**Supplemental Figure 4. Central Macular Thickness**


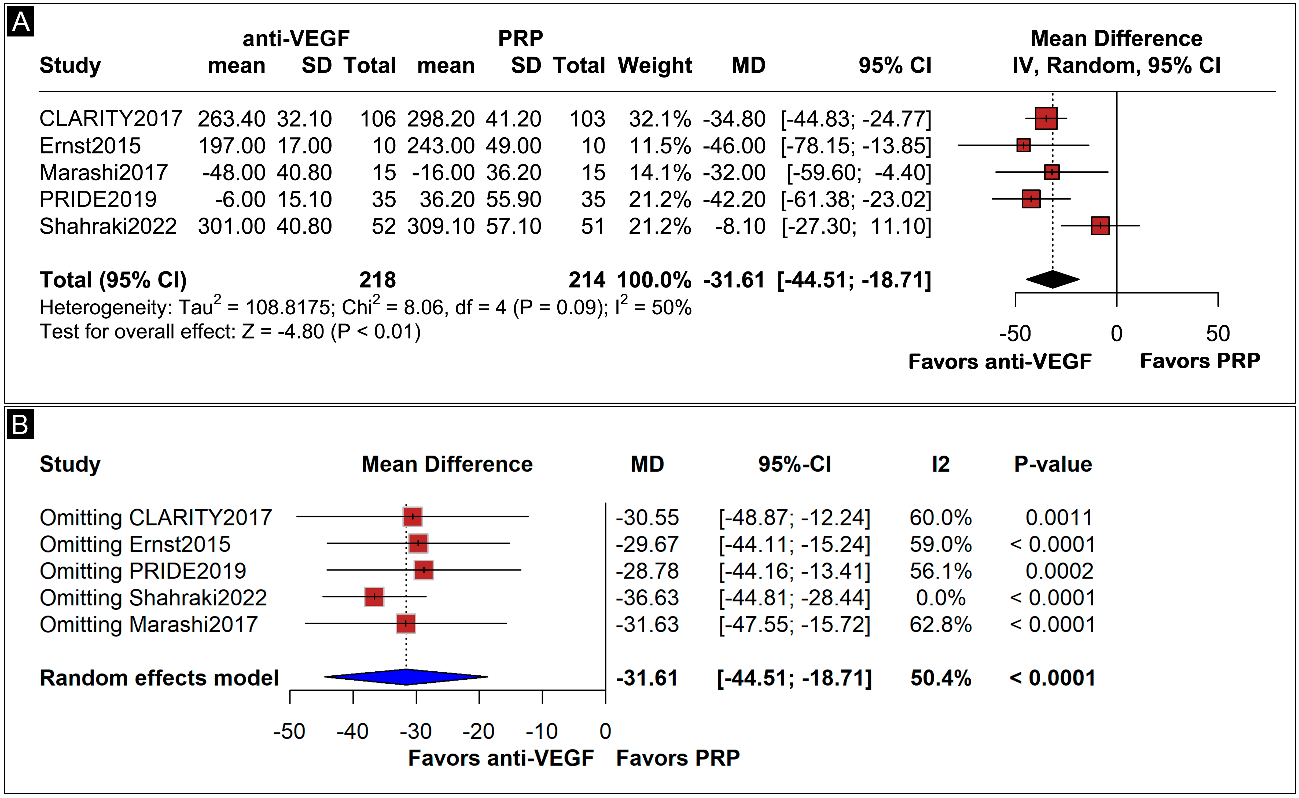


Supplemental Figure 4. (A) Forest plot comparing central macular thickness (CMT) between anti-VEGF and PRP groups, showing a significant reduction favoring anti-VEGF (MD -31.61 µm; 95% CI -44.51, -18.71; p < 0.01; I² = 50%) in the short-term. (B) Leave-one-out sensitivity analysis indicating that Shahraki and colleagues influenced heterogeneity, reducing it to 0% upon exclusion without altering the overall effect.

**Supplemental Figure 5. Diabetic Macular Edema**


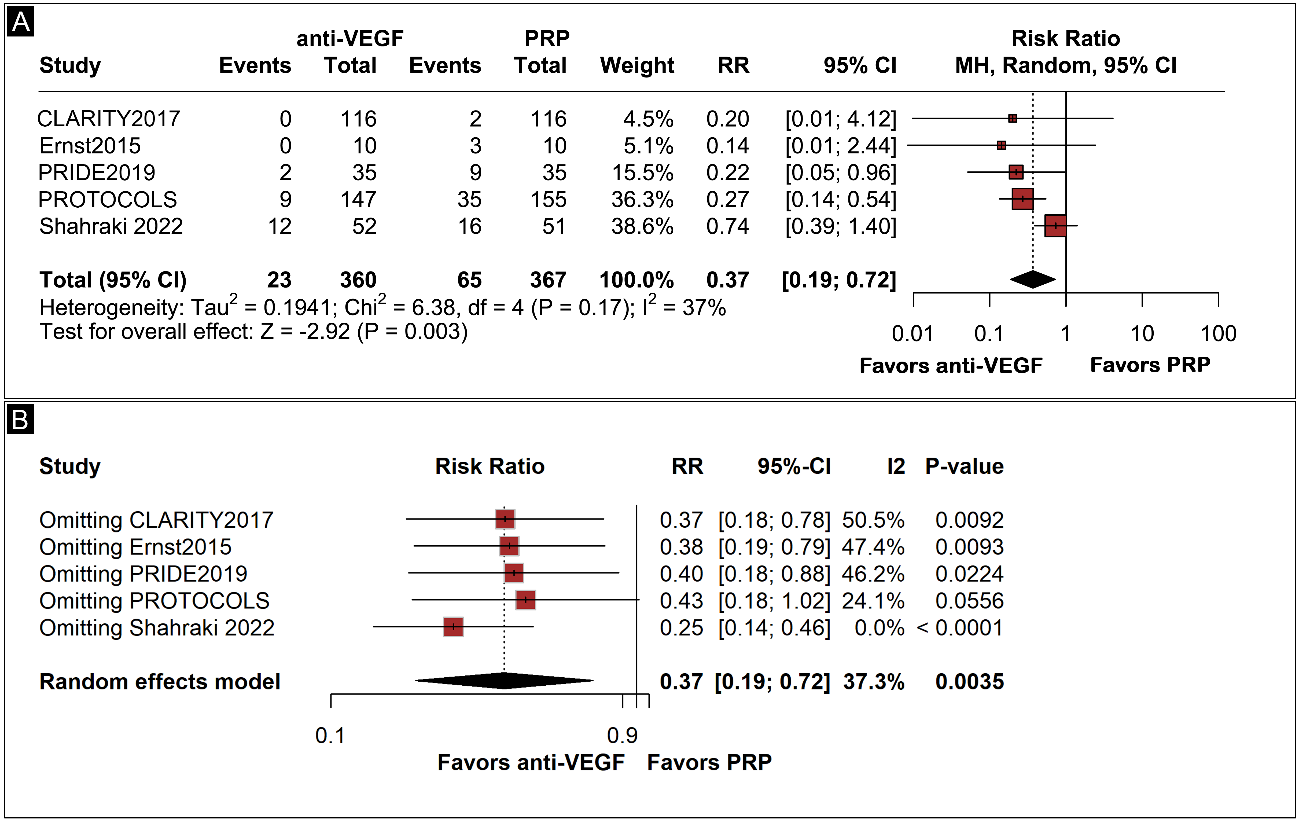


Supplemental Figure 5. (A) Forest plot showing a significant reduction in diabetic macular edema (DME) rates favoring anti-VEGF over PRP (RR 0.37; 95% CI 0.19, 0.72; p = 0.003; I² = 37%) in the short-term. (B) Leave-one-out sensitivity analysis reveals that Shahraki and colleagues had a notable impact, reducing heterogeneity to 0% upon exclusion, but the overall effect remained consistent.

**Supplemental Figure 6. Short term tractional retinal detachment rates**


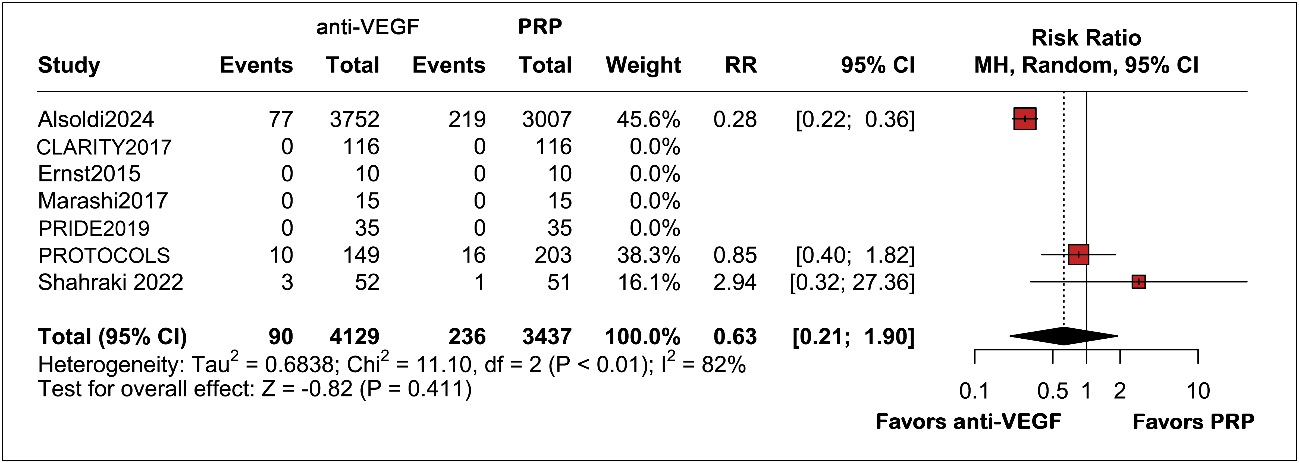


Supplemental Figure 6. Forest plot showing short-term tractional retinal detachment (TRD) rates, with no significant difference between anti-VEGF and PRP groups (RR 0.63; 95% CI 0.21, 1.90; p = 0.411; I² = 82%). High heterogeneity indicates substantial variability between studies.

**Supplemental Figure 7. Thromboembolic events**


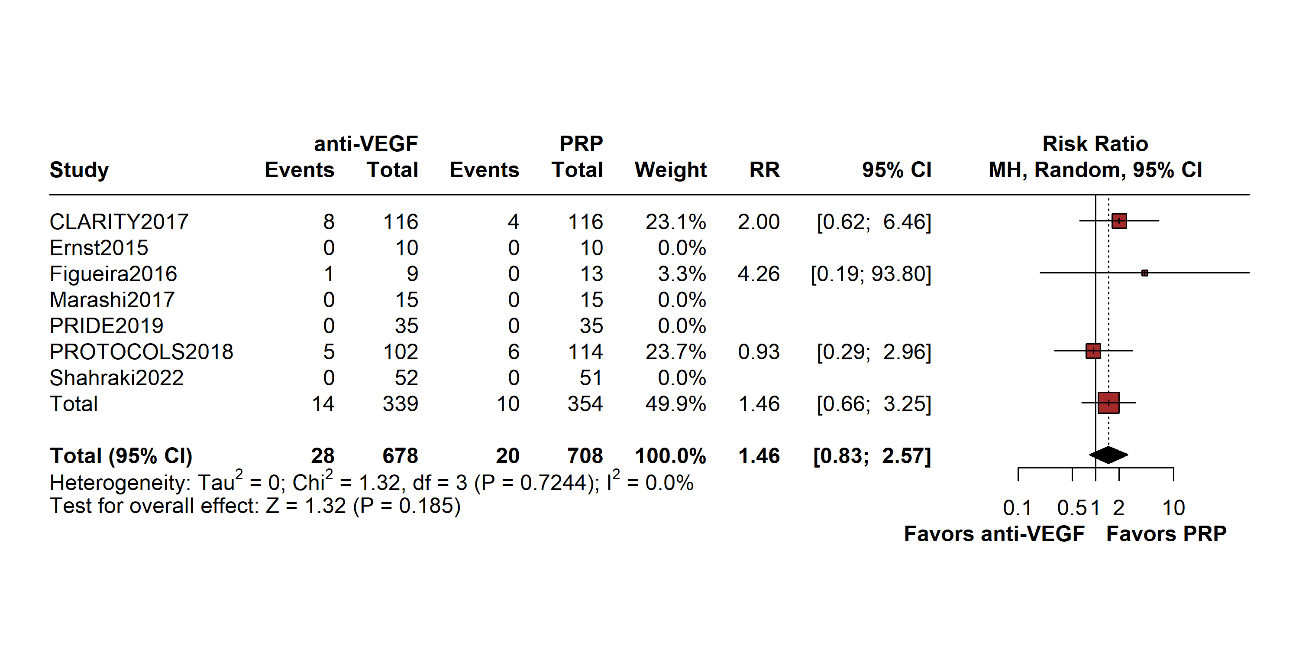


Forest plot comparing the risk ratio (RR) of events in the anti-VEGF group versus the PFC (panretinal photocoagulation) group across multiple studies. The plot shows the individual study estimates (with 95% confidence intervals) and the overall pooled estimate (RR = 1.46, 95% CI [0.66, 3.24]), indicating no significant difference between the groups. The size of the squares represents the weight of each study in the meta-analysis, while the diamond indicates the overall effect estimate with its confidence interval. A risk ratio (RR) greater than 1 suggests a higher risk in the anti-VEGF group, while an RR less than 1 favors PFC. The heterogeneity among the studies is low (I² = 0%), indicating consistency in the results

| **Study** | **Journal** | **Type of study** | **Location** | **Source of founding** | **Patients/eyes** | **PRP/Anti-VEGF** | **Male/**  **Female** | **Mean age (years old)** | **Anti-VEGF** | **Anti-VEGF Scheme** | **LASER** | **HbA1c PRP/Anti-VEGF** | **DM PRP** | **DM Anti-VEGF** | **Included DME** | **Follow-up** | **Patients with DME** |
| --- | --- | --- | --- | --- | --- | --- | --- | --- | --- | --- | --- | --- | --- | --- | --- | --- | --- |
| **Alssoudi 2024** | JAMA Ophthalmology | Retrospective cohort study | Multiple centers (107 health care organizations across 15 countries, including 60 health care organizations within the US) | Genentech | 12.040/12.040 | 6020 / 6020 | 11.572 / 11.672 | PRP: 64.8 // Anti-VEGF: 66.1 | N/A | N/A | PRP | 11.9 / 11.8 | N/A | N/A | N/A | 5 years | N/A |
| **ClARITY 2017** | THE LANCET | RCT | United Kingdom | EME | 232 / 232 | 116 / 116 | 155 / 77 | PRP: 50.8 // Anti-VEGF: 51.5 | Aflibercept | As needed | PRP | <8% (44), 8-10% (48), >10% (24) // <8% (41), 8-10% (51), >10% (24) | Type 1 (51), Type 2 (65) | Type 1 (54), Type 2 (62) | Yes | 1 year | 59/232 eyes |
| **Ernst 2012** | Acta Ophthalmologica 2012 | RCT | USA | N/A | 10 / 20 | 10 / 10 | 3 / 7 | 53 ± 9 for both | Bevacizumab | Fixed | PRP | N/A | Only Type 2 | Only Type 2 | N/A | 1 year | N/A |
| **Figueira 2016** | Ophthalmologica | RCT | Portugal | Novartis | 22 / 22 | 13 / 9 | 16 / 7 | PRP: 54 // Anti-VEGF: 61 | Ranibizumab | As needed | PRP | 8 / 7.55 | Only Type 2 | Only Type 2 | N/A | 1 year | N/A |
| **Marashi 2017** | Investigative Ophthalmology & Visual Science | RCT | Syria and Jordan | N/A | 30 / 30 | 15 / 15 | 7 / 23 | PRP: 53 // Anti-VEGF: 52 | Bevacizumab | Fixed | PRP | N/A | N/A | N/A | Yes | 1 year | 15/30 eyes |
| **PRIDE 2020** | Acta Ophthalmologica 2020 | RCT | Multicenter study | Novartis Pharma Gmbh, Allergan, Alcon Pharma GmbH, Bayer AG, Boehringer Ingelheim, Carl Zeiss Meditec, Alimere Sciences | 70 / 70 | 35 / 35 | 45 / 25 | PRP: 53 // Anti-VEGF: 52.5 | Ranibizumab | As needed | PRP | 8.1 / 8.2 | Type 1 (17), Type 2 (18) | Type 1 (15), Type 2  (20) | No | 1 year | 0 |
| **PROTOCOLS 2018** | JAMA Ophthalmology | RCT | USA | Genetech | 305 / 394 | 203 / 191 | N/A | 52 for both | Ranibizumab | As needed | PRP | N/A | N/A | N/A | Yes | 5 years | 88/394 eyes |
| **Shahraki 2022** | THE JOURNAL OF RETINAL AND VITREOUS DISEASES | RCT | Iran | N/A | 103 / 130 | 51 / 52 | 53 / 50 | PRP: 53.52 // Anti-VEGF: 51.96 | Bevacizumab | Fixed | PRP | 8.54 / 8.42 | Only Type 2 | Only Type 2 | Yes | 1 year | 91/153 eyes |

**Table 1**. Baseline characteristic of included studies

Anti-VEGF: anti-vascular endothelial growth factor

DM: diabetes mellitus

DME: diabetic macular edema

HbA1c: hemoglobin A1C

PRP: panretinal photocoagulation

RCT: randomized clinical trial

N/A: not available

|  |  | | Summary Table on Long-term Studies | |  | |
| --- | --- | --- | --- | --- | --- | --- |
|  | Long-term TRD rates and conclusions | | Long-term VH rates and conclusions | | Long-term PPV rates and conclusions | |
| Alsoudi et al | - **Anti-VEGF monotherapy:** 2.4% (126 events) - **PRP monotherapy:** 6.7% (347 events) - **Risk Ratio (RR):** 2.76 (95% CI 2.26-3.37) | *“These findings support the hypothesis that* ***patients with PDR***  ***treated with PRP monotherapy*** *are more likely to develop VH,* ***TRD,*** *and undergo PPV when*  *compared with matched patients treated with anti-VEGF monotherapy.”* | - **Anti-VEGF monotherapy:** 367 events (9.8%) - **PRP monotherapy:** 506 events (16.8%) - **RR:** 1.72 (95% CI 1.52-1.95) | *“Patients with PDR treated with PRP monotherapy* ***are more likely to develop VH.****”* | - **PRP monotherapy:** 548 events (9.1%) - **Anti-VEGF monotherapy:** 465 events (7.7%) - **RR:** 1.18 (95% CI 1.05-1.36) | *“In conclusion****, this study revealed increased risks of requiring***  ***vitrectomy in patients with PDR treated with PRP monotherapy***  ***when compared with patients with PDR treated with***  ***anti-VEGF monotherapy*** *at follow-ups of 1 year, 3 years,* ***and 5***  ***years*** *in a large patient dataset within the clinical practice setting.”* |
| PROTOCOL S | - **Ranibizumab group:** 5.0% (10 eyes) - **PRP group:** 12.0% (24 eyes) - The study did not provide a measure of association for this outcome | *“Severe vision loss or serious complications of PDR, such as neovascular glaucoma, iris NV, or* ***macular traction retinal*** *detachments****, were uncommon with PRP or ranibizumab.”*** | - **Ranibizumab group:** 48.0% (91 eyes) - **PRP group:** 46.0% (93 eyes) - Vitreous hemorrhage developed in 91 eyes in the ranibizumab group and 93 in the PRP group (cumulative probabilities, 58% vs 54%; adjusted difference, 4% [95% CI, −7% to 16%]; P = .47)." | “There was **no difference in VH** rates between the two groups.” | - **PRP monotherapy:** 39 events (22%) - **Anti-VEGF monotherapy:** 21 events (15%) - **Hazard ratio:** 0.5 (95% CI 0.3-0.8) | The authors have not discussed thoroughly the difference in rates of PPV between groups. They mentioned low rates of serious complications of PDR.  *“Severe vision loss or serious complications of PDR, such as neovascular glaucoma, iris NV, or* ***macular traction retinal*** *detachments****, were uncommon with PRP or ranibizumab.****”* |
| Meta-analyse | - **Anti-VEGF monotherapy:** 3.4% (136 events) - **PRP monotherapy:** 11.5% (371 events) - **RR:** 0.31 (95% CI 0.23, 0.42) - **GRADE assessment:** high certainty of evidence. | *“Regarding longer-term outcomes, we observed a significantly higher rate of TRD in patients within the PRP group compared to those in the anti-VEGF group, with a high certainty of evidence.* ***Specifically, patients treated with anti-VEGF therapy had a 69% lower risk of developing this severe complication****. “* | - **Anti-VEGF monotherapy:** 11% (458 events) - **PRP monotherapy:** 18% (599 events) - **RR:** 0.77 (95% CI 0.43, 1.38; I² = 96%) - **GRADE assessment:** low certainty of evidence. | *“Our findings regarding VH rates were* ***inconclusive****”* | - **Anti-VEGF monotherapy:** 7.8% (486 events) - **PRP monotherapy:** 9.4% (587 events) - **RR:** 0.75 (95% CI 0.53-1.07) | *“Pooled data on PPV rates showed* ***no significant difference*** *between the groups.”* |

**Abbreviations:**

**Anti-VEGF –** Anti-Vascular Endothelial Growth Factor**; CI –** Confidence Interval**; GRADE –** Grading of Recommendations, Assessment, Development, and Evaluations**; I² –** I-squared statistic (measure of heterogeneity)**; NV –** Neovascularization**; PDR –** Proliferative Diabetic Retinopathy**; PPV –** Pars Plana Vitrectomy**; PRP –** Panretinal Photocoagulation**; RR –** Risk Ratio; **TRD** – Tractional Retinal Detachment; **VH** – Vitreous Hemorrhage;

**Competing Interests Statement:**

The authors declare that they have no competing interests.
